# NS1-binding protein radiosensitizes esophageal squamous cell carcinoma by transcriptionally suppressing c-Myc

**DOI:** 10.1186/s40880-018-0307-y

**Published:** 2018-06-05

**Authors:** Yuwen Wang, Jingjing Cheng, Dan Xie, Xiaofeng Ding, Hailing Hou, Xi Chen, Puchun Er, Furong Zhang, Lujun Zhao, Zhiyong Yuan, Qingsong Pang, Ping Wang, Dong Qian

**Affiliations:** 1 Department of Radiotherapy, Tianjin Medical University Cancer Institute and Hospital, Tianjin, 300060 P. R. China; 2 National Clinical Research Center for Cancer, Tianjin Medical University Cancer Institute and Hospital, Tianjin, 300060 P. R. China; 3 Key Laboratory of Cancer Prevention and Therapy, Tianjin, Tianjin’s Clinical Research Center for Cancer, Huanhu West Street, Tianjin, 300060 P. R. China; 4 State Key Laboratory of Oncology in South China, Guangzhou, 510060 Guangdong P. R. China; 5 Sun Yat-sen University Cancer Center, Guangzhou, 510060 Guangdong P. R. China

**Keywords:** Esophageal squamous cell carcinoma, NS1-BP, Prognostic biomarker, Radiotherapy, c-Myc

## Abstract

**Background:**

Cisplatin-based chemotherapy with concurrent radiotherapy is a standard treatment for advanced esophageal squamous cell carcinoma (ESCC). NS1-binding protein (NS1-BP), a member of the BTB-kelch protein family, has been shown to inhibit the proliferation of Hela cells by suppressing c-Myc. In the present study, we examined the potential function role of NS1-BP expression in ESCC, and particularly, the sensitivity of ESCC to radiotherapy.

**Methods:**

NS1-BP expression was examined using immunohistochemistry in two cohorts (n = 98 for the training cohort; n = 46 for independent validation cohort) of ESCC patients receiving cisplatin-based chemotherapy and concurrent radiotherapy. Normal esophageal mucosal tissue blocks were used as a control. We also conducted a series of in vitro and in vivo experiments to examine the potential effects of over-expressing NS1-BP on ESCC cells, and particularly their sensitivity to ionizing irradiation.

**Results:**

In the training cohort, NS1-BP downregulation was observed in 59% (85/144) of the ESCC specimens. NS1-BP downregulation was associated with chemoradiotherapeutic resistance and shorter disease-specific survival (DSS) in both the training and validation cohorts. Over-expressing NS1-BP in cultured ESCC cells substantially increased the cellular response to irradiation both in vitro and in vivo. NS1-BP also significantly enhanced IR-induced apoptosis, and abrogated IR-induced G_2_/M cell-cycle arrest and ATM/Chk1 phosphorylation. Immunoprecipitation assays indicated that NS1-BP could interact with *c*-*Myc* promoter regions to inhibit its transcription. In ESCC tissues, c-Myc expression was inversely correlated with NS1-BP levels, and was associated with a shorter DSS.

**Conclusions:**

Our findings highlight the role and importance of NS1-BP in radiosensitivity of ESCC. Targeting the NS1-BP/c-Myc pathway may provide a novel therapeutic strategy for ESCC.

## Background

Esophageal carcinoma is the eighth most common cancer worldwide and the sixth leading cause of cancer-related mortality [1–3]. Esophageal squamous cell carcinoma (ESCC) is the most common histological type of esophageal carcinoma, with a 5-year overall survival of only 15%–25% [1–3]. Most ESCC patients present with locally advanced disease. Radiotherapy is a critical part of the overall treatment of ESCC. Neo-adjuvant or definitive chemoradiotherapy is the standard therapeutic modality. However, ESCC patients exhibit variable responses to radiotherapy and currently available diagnostic modalities do not accurately predict the clinical response to radiotherapy [4, 5].

The BTB-kelch protein family consists of a group of approximately 50 proteins sharing an *N*-terminal broad complex, tramtrack, and bric-a-brac (BTB) or poxvirus zinc finger domains and *C*-terminal kelch repeats. The BTB domain is a protein–protein interaction motif, and the kelch repeats form a β-propeller structure that is involved in protein–protein interaction. Based on this structure, most BTB-kelch proteins function as substrate adapters, recruiting proteins destined for ubiquitination to the cullin-RING E3 ubiquitin ligases, and enable the identification of a wide range of substrates for ubiquitination [6]. Some of the BTB-kelch proteins regulate the actin cytoskeleton, thereby affecting cell–cell and cell–substrate interactions, and cell migration [7, 8]. Krem et al. [9] reported that the BTB-kelch protein KLHDC8B protects against mitotic errors and chromosomal instability, and is essential for mitotic regulation and chromosomal segregation in Hodgkin lymphoma.

NS1-BP belongs to the BTB-kelch protein family, and interacts with NS1. NS1 is the only non-structural protein of influenza A viruses, and specifically induces viral mRNA expression, thus acting as a powerful translational enhancer [10, 11]. NS1 interacts with NS1-BP to inhibit NS1 pre-mRNA splicing and to influence influenza A viral gene expression and replication [12, 13]. However, the role of NS1-BP in influenza A virus infection remains unclear. Up to now, only one study has investigated the role of NS1-BP in cancer cells; Perconti et al. [14] reported that NS1-BP inhibited the proliferation of HeLa cells by suppressing c-Myc at the transcriptional level. Moreover, ectopic overexpression of NS1-BP increased the repression of basal *c*-*Myc* transcription, and disrupted steady state levels of endogenous c-Myc mRNA and protein [14]. However, the clinical significance of NS1-BP has not been well established in human cancers.

c-Myc is a highly pleiotropic transcription factor that controls cell cycle progression, proliferation, growth, adhesion, differentiation, apoptosis, and metabolism [15, 16]. Aberrant c-Myc expression is widely implicated in tumorigenesis, sustained tumor growth and drug resistance in many tumor types [17, 18]. c-Myc also increases resistance of tumor cells to irradiation by regulating downstream genes such as cyclin-dependent kinase 4 (*CDK4*), ataxia-telangiectasia mutated kinase (*ATM*), checkpoint kinase 1 (*Chk1*), and *Chk2* [19]. Therefore, NS1-BP may affect tumorigenesis and determine cellular chemo- and radio-sensitivity via regulation of c-Myc.

Here, we investigated the expression of NS1-BP in ESCC, and tested its possible role as a prognostic biomarker for ESCC patients treated with chemoradiotherapy. We also conducted a series of experiments using ESCC cell lines to explore the potential effects of NS1-BP in vitro and in vivo.

## Materials and methods

### Acquisition of tissue specimens

The training cohort consisted of 98 patients with advanced ESCC with paraffin-embedded tissue archived at Sun Yat-sen University Cancer Center (Guangzhou, China) between 2002 and 2008. Thirty healthy esophageal mucosa tissue blocks were retrieved as the control. The validation cohort consisted of 46 patients with advanced ESCC receiving treatment at the Tianjin Medical University Cancer Institute and Hospital (Tianjin, China). All tissue specimens were obtained as diagnostic biopsies via esophagoscopy and pathologically confirmed before initiation of any antitumor therapy. All patients received cisplatin-based chemotherapy and concurrent radiotherapy (daily dose of 1.8–2.0 Gy to a total dose of 60–70 Gy over 6–7 weeks). In addition, 10 paired fresh ESCC tissues and adjacent non-neoplastic esophageal mucosa tissues were collected at Tianjin Medical University Cancer Institute and Hospital. ESCC was staged according to the 6th edition of the International Union against Cancer (UICC 2002).

The study protocol was approved by the Ethics Committees at Sun Yat-sen University Cancer Center and Tianjin Medical University Cancer Institute and Hospital. Written informed consent was obtained from all patients. Patient data were anonymized.

### Patient evaluation

Starting from 4 weeks after chemoradiotherapy, patients were evaluated every 3 months for the 1st year and then every 6 months for the next 2 years, and thereafter annually according to the World Health Organization (WHO) criteria. The diagnostic examinations consisted of esophagography, computed tomography (CT), chest X-ray, abdominal ultrasonography and bone scan, when necessary, to detect tumor recurrence and/or metastasis. Complete response (CR) was defined as no evidence of disease on imaging and complete resolution of all assessable lesions by endoscopic biopsy. Partial response (PR) was defined as a 30% or greater reduction in tumor maximum dimension and no progression of assessable lesions. Stable disease (SD) was defined by a reduction by < 50% or increase < 25% in tumor size. All these conditions had to last for at least 4 weeks and there was no appearance of new lesions. Progressive disease (PD) was defined as an increase ≥ 25% in tumor size or the appearance of new lesions.

### Cells

Human ESCC cell lines KYSE30, KYSE510, KYSE410, and KYSE140 (South China State Key Laboratory of Oncology, Sun Yat-sen University), and TE-1 (Cell Resource Center, Shanghai Institutes for Biological Sciences, Chinese Academy of Sciences), and primary cultured esophageal squamous epithelial cells (South China State Key Laboratory of Oncology) were used in the current study. KYSE30, KYSE150, KYSE410, and KYSE140 were maintained in RPMI-1640 (Gibco, Buffalo, Grand Island, NY, USA) and TE-1 in DMEM, supplemented with 10% fetal bovine serum (Gibco) and 1% penicillin–streptomycin at 37 °C in a 5% CO_2_ incubator. KYSE30 and TE-1 were authenticated by short tandem repeat fingerprinting at China Center for Type Culture Collection (CCTCC, Wuhan University, Wuhan, China). Radiation was delivered using 320 kV X-ray machine (Precision X Ray Inc.) at a dose rate of 2.3 Gy/min.

### Immunohistochemistry

Paraffin-embedded tissue blocks were cut into 4-μm-thick sections, and dewaxed using xylene, followed by rehydration through gradient ethanol. Antigen retrieval was carried out by heating in a microwave. Tissue slides were immersed in 3% hydrogen peroxide for 15 min. Nonspecific binding was blocked by normal goat serum. Tissue slides were incubated overnight with anti-NS1-BP antibody (1:500, Abcam) at 4 °C, and then rinsed with phosphate buffered saline (PBS). Subsequently, the sections were incubated with a secondary antibody for 40 min at 37 °C, washed with PBS, and then stained with diaminobenzidine, followed by counter staining with hematoxylin.

NS1-BP expression was assessed by two independent pathologists who were blinded to patient data. Upon disagreement, the two pathologists reassessed the results until a consensus was reached. A staining index obtained as the intensity of positive staining (moderate low, 1; moderate high, 2; strong, 3) and the proportion of immune-positive cells of interest (0%–25%, 1; 25%–50%, 2; 50%–75%, 3; 75%–100%, 4) was calculated. The two scores were multiplied to yield the total score (1, 2, 3, 4, 6, 8, 9, and 12). Expression level was classified into either low (score 1–4) or high (score 6–12).

### Western blotting assays

Antibodies against the following proteins were used for immunoblotting assays: NS1-BP, survivin and P27 (Abcam, Cambridge, MA, USA), c-Myc, cleaved-caspase3, cleaved-poly(ADP-ribose) polymerase (PARP), cyclin-dependent kinase (CDK) 4, phospho-ATM (Ser1981), phospho-Chk1 (Ser345) and phospho-Chk2 (Thr68) (Cell Signalling Technology, Danvers, MA, USA), and GAPDH (Santa Cruz Biotechnology, Santa Cruz, CA, USA). GADPH was used as the loading control. Protein concentration was detected using a BCA kit.

### MTT assays

Cell viability was measured by a commercially available 3-(4,5-dimethylthiazol-2-yl)-2,5-diphenyl tetrazolium bromide (MTT) assay (Sigma, St. Louis, MO, USA). Briefly, cells were seeded in 96-well plates, and then cultured for 4 h after addition of MTT. Optical density (OD) was measured at 490 nm using a microplate reader. The test was repeated 3 times independently.

### Flow cytometry

For analysis of apoptosis, ESCC cells were stained by annexin V-FITC and propidium iodide to determine the percentage of apoptotic cells using a kit from BD Biosciences (Bedford, MA, USA). Each sample was then analyzed with flow cytometry (BD FACSCanto II Flow Cytometer; BD Biosciences).

For cell cycle analysis, cells were washed twice with PBS, and fixed in 70% ethanol overnight at 4 °C. The fixed cells were pelleted by centrifugation at 2000×*g* for 10 min, re-suspended in PBS, stained with 10 mg/mL propidium iodide for 5 min, and incubated in the dark at 4 °C for 30 min. The DNA content was determined using a flow cytometer (Model 100, Beckman Coulter, Kraeme, CA, USA) with excitation and emission settings of 488 and 610 nm, respectively. The proportion of G_2_/M cells was calculated based on the relative DNA content, as measured by the flow cytometer using LYSIS software (Becton–Dickinson).

### Clonogenic assays

Survival following ionizing irradiation (IR) was assessed for the ability of cells to maintain their clonogenicity. Briefly, after IR, KYSE30 and TE-1 ESCC cells were trypsinized, counted, and seeded for colony formation in 6-well plates with 50–5000 cells per well. After incubation intervals of 14–21 days, colonies were stained with crystal violet and manually counted. An aggregate consisting of 50 cells or more was considered a colony and scored, and 5 replicate wells containing 10–150 colonies per well were counted for each treatment. Experiments were done in triplicate.

### Plasmid construction, lentivirus production and transduction

NS1-BP cDNA was cloned into a pCDH cDNA expression lentivector (System Biosciences; Mountain View, CA, USA). For loss-of-function study, plasmid containing a validated short hairpin RNA (shRNA) targeting *NS1*-*BP* was cloned into the vector pLLU2G, which is derived from pLL3.7 and contains separate GFP and shRNA expression elements as well as elements required for lentiviral packaging [20]. The target sequence of *NS1*-*BP* for constructing lentiviral shRNA was 5′-CCAGCAATGGCAAATTATA-3′ (shNS1-BP). The cDNA of NS1-BP construct and shRNA were purchased from Genechem (Shanghai, China). The lentiviral expression construct and packaging plasmid mix were co-transfected into 293 cells to generate the recombinant lentiviruses.

### Immunofluorescence

Cells grown on coverslips were fixed in 4% paraformaldehyde. Fixed cells were permeabilized with 1% Triton-100 and blocked with 10% normal goat serum. Cells were then incubated with a primary antibody against γ-H2AX (Cell Signalling) for 2 h at 37 °C in a humidified chamber, followed by incubation with a secondary antibody for 1 h. Immunofluorescence images were captured using with FV10-ASW viewer software (Olympus, Tokyo, Japan).

### Luciferase assays

For dual luciferase reporter assays, 5 × 10^4^ cells were seeded into each well of 24-well plates. A firefly luciferase reporter construct under the control of the *c*-*Myc* promoter (1 μg) and a Renilla luciferase reporter construct under the control of the *TK* promoter for normalization of transfection efficiency (10 ng) were co-transfected into cells in triplicate using FuGENE6 (Roche Diagnostics, Basle, Switzerland) at a ratio of 1 μg of plasmid to 3 μL of FuGENE6. Luciferase activity was examined at 24 h post transfection using the Dual-Luciferase Assay kit (Promega, Beijing, China) and normalized to firefly luciferase activity. Experiments were performed at least three times independently and each combination was tested in triplicate.

### Chromatin immunoprecipitation assays

Chromatin immunoprecipitation (ChIP) assays were performed by using a kit from Merck Millipore (Darmstadt, Germany), with minor modifications [21]. The NS1-BP antibody (Abcam) and protein A/G PLUS-Agarose (Santa Cruz Biotechnology) were used. In human cells, four promoters, P0, P1, P2, and P3, of *c*-*Myc* have been documented, with P2 being the maximally used promoter [15, 22]. NS1-BP is one of the alpha-enolase/MBP-1 partners, and binds to the P2 promoter of *c*-*Myc* specifically [14, 23]. Herein, the P2 promoter was used in the ChIP assays, and the P0 promoter was used as a non-related *c*-*Myc* promoter negative control. The primer sequences used for the ChIP assays were as follows: P2, forward, 5′-AGGCGCGCGTAGTTAATTCAT-3′ and reverse, 5′-CGCCCTCTGCTTTGGGA-3′; P0, forward, 5′-CCCAACAAATGCAATGGGAGTTTATTCA-3′ and reverse, 5′-TCAAGAGTCCCAGGGAGAGTGGAGG-3′.

### Xenograft assays

Four-week-old male severe combined immunodeficient (SCID-Beige) mice were injected subcutaneously with 2 × 10^5^ TE-1-NS1-BP or TE-1-NS1-BP-control cells. When the tumor mass became palpable (about 120 mm^3^), mice were randomly divided into a control group and an IR group (n = 6/group). Effects of IR (6 Gy, 2 Gy/fraction every other day for 3 days) were monitored. Tumor volume (V) was determined by measuring the length (L) and width (W) of the tumor with a caliper and calculated using the formula V = (L × W^2^) × 0.5. All the procedures were carried out in accordance with the guidelines of the laboratory animal ethics committee of Tianjin Medical University Cancer Institute and Hospital.

### Statistical analysis

Statistical analysis was performed using SPSS 16.0 (SPSS Inc., Chicago, IL, USA). Data are expressed as mean ± standard error (SE) and analyzed with two-sided Student’s *t* test. χ^2^ test was used to analyze the association between NS1-BP expression and clinicopathologic features of ESCC patients. Survival data were analyzed using the Kaplan–Meier analysis followed by the log-rank test. Univariate and multivariate survival analysis were done using the Cox-regression model. Statistical significance was set at *P* < 0.05.

## Results

### NS1-BP is downregulated in ESCC tissues

**Fig. 1:**
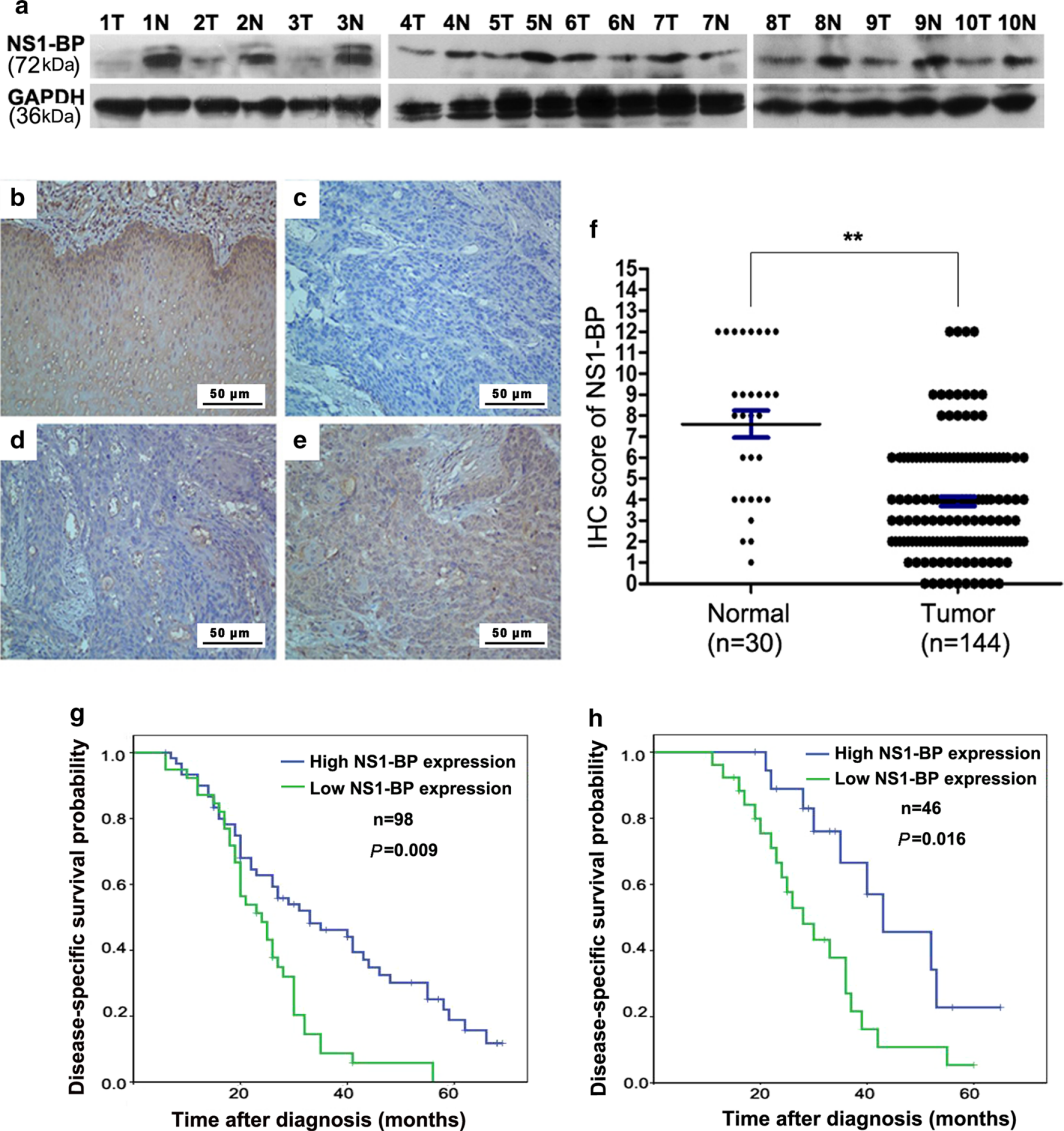
NS1-BP expression in esophageal squamous cell carcinoma (ESCC) and its prognostic significance in ESCC patients. **a** Western blot showing lower NS1-BP levels in ESCC tissues than adjacent non-neoplastic esophageal mucosa tissues (ANT) in 8 of 10 cases. **b** Normal esophageal mucosa specimen showing strong staining of NS1-BP (IHC score = 12). **c** ESCC sample (case 15) exhibiting negative NS1-BP staining. **d** ESCC sample (case 8) exhibiting low NS1-BP expression (IHC score = 3). **e** ESCC sample (case 60) exhibiting high NS1-BP expression (IHC score = 9). **f** Statistical analysis of significantly low expression of NS1-BP in ESCC tissues (**P < 0.01). **g**, **h** Low expression of NS1-BP was associated with poor prognosis of ESCC patients. Kaplan–Meier plots showing disease-specific survival in 98 ESCC patients in the training cohort (**g**) and 46 ESCC patients in the validation cohort (**h**), according to NS1-BP expression levels in the primary tumor (*P* = 0.009 and 0.016, log-rank test)

Protein expression was first examined by Western blotting assays in 10 paired fresh ESCC and adjacent normal esophageal tissues. NS1-BP levels were lower in ESCC tissues than adjacent normal tissues in 8 cases (Fig. 1a). Immunostaining revealed lower NS1-BP expression in the primary lesions in the training cohort that consisted of 144 patients with advanced ESCC versus 30 normal esophageal mucosa tissues (Fig. 1b–f). Based on the immunohistochemistry evaluation, 59% (85/144) of the ESCC tumor tissues had low NS1-BP expression. In contrast, only 30% (9/30) of normal esophageal mucosa tissues had low NS1-BP expression (*P *= 0.012). NS1-BP levels were significantly lower in tumor tissues than normal esophageal mucosa tissues (Fig. 1f, *P* < 0.01).

### Downregulated NS1-BP predicts chemoradiotherapeutic resistance

Treatment responses in the training cohort consisted of CR (19/98), PR (42/98), SD (36/98) and PD (1/98). In the validation cohort, treatment responses included CR (10/46), PR (19/46), SD (16/46) and PD (1/46). Of the 115 patients who did not achieve a CR, 31 received adjuvant chemotherapy, and four underwent radical esophagectomy. The remaining patients did not receive any anti-tumor treatments until tumor progression. Low NS1-BP expression was positively associated with resistance to chemoradiotherapy in both the training cohort (*P* = 0.007) and the validation cohort (*P *= 0.003; Table 1). No correlation was observed between NS1-BP expression and other clinicopathologic variables (*P *> 0.05; Table 1).

**Table 1:** Association between clinicopathologic factors and NS1-BP expression in esophageal squamous cell carcinoma (ESCC) patients

Variables	Learning cohort [cases (%)]			Validation cohort [cases (%)]		
	High expression	Low expression	P*	High expression	Low expression	P*
Age (years)			0.775			0.547
≤ 55^a^	22 (40.7)	32 (59.3)		10 (40.0)	15 (60.0)	
> 55	19 (43.1)	25 (56.9)		8 (38.0)	13 (62.0)	
Gender			0.501			0.709
Male	34 (41.5)	48 (58.5)		15 (36.8)	23 (63.2)	
Female	7 (43.8)	9 (56.2)		3 (37.5)	5 (62.5)	
WHO grade			0.719			0.660
G1	11 (45.8)	13 (54.2)		4 (36.4)	7 (63.6)	
G2	20 (40.0)	30 (60.0)		8 (40.0)	12 (60.0)	
G3/4	10 (41.7)	14 (58.3)		6 (40.0)	9 (60.0)	
Tumor size (cm)			0.640			0.387
≤ 6^b^	22 (39.3)	34 (60.7)		13 (40.7)	19 (59.3)	
> 6	19 (45.2)	23 (54.8)		5 (35.8)	9 (64.2)	
T category			0.541			0.174
T2	5 (41.7)	7 (58.3)		6 (50.0)	6 (50.0)	
T3	14 (40.0)	21 (60.0)		6 (33.3)	12 (66.7)	
T4	22 (43.1)	29 (56.9)		6 (37.5)	10 (62.5)	
N category			0.622			0.328
N0	8 (47.1)	9 (52.9)		6 (42.9)	8 (57.1)	
N1	33 (40.8)	48 (59.2)		12 (37.5)	20 (62.5)	
M category			0.352			0.302
M0	27 (45.8)	32 (54.2)		10 (41.7)	14 (58.3)	
M1-lym	14 (35.9)	25 (64.1)		8 (31.9)	14 (68.1)	
CRT response			0.007			0.003
CR	14 (73.7)	5 (26.3)		8 (80.0)	2 (20.0)	
Not CR	27 (34.2)	52 (65.8)		10 (27.8)	26 (72.2)	

*ESCC* esophageal squamous cell carcinoma, *T* tumor, *N* node, *M* metastases, *M1-lym* distant lymph node metastasis, *CR* complete response

* χ^2^ test

^a^Mean age

^b^Mean tumor size

### NS1-BP downregulation predicts a shorter DSS

Univariate analysis revealed significant differences in disease-specific survival (DSS) between gender, WHO grade, TNM category, treatment response, and NS1-BP expression (Table 2). Low NS1-BP expression was associated with shorter DSS in the training cohort (median 18.6 versus 32.6 months, *P* = 0.019; Fig. 1g) as well as in the validation cohort (median 15.3 versus 33.5 months, *P* = 0.016; Fig. 1h). Multivariate analysis revealed that NS1-BP expression and treatment response were independent predictors of DSS in the training cohort (*P* = 0.019 and 0.003, respectively, Table 3) as well as in the validation cohort (*P *= 0.010 and 0.008, respectively, Table 3).

**Table 2:** Univariate Cox regression analysis of potential prognostic factors for ESCC patients

Variables	Learning cohort		Validation cohort	
	HR (95% CI)	P*	HR (95% CI)	P*
Age (years)				
≤ 55^a^	1.000		1.000	
> 55	1.075 (0.743–1.398)	0.826	1.115 (0.673–1.375)	0.747
Gender				
Male	1.000		1.000	
Female	0.673 (0.512–0.887)	0.036	0.596 (0.425–0.862)	0.041
WHO grade				
G1	1.000		1.000	
G2	1.376 (0.373–3.746)	0.019	1.542 (0.538–4.137)	0.033
G3/4	2.479 (0.469–4.837)	< 0.001	2.058 (0.558–6.726)	< 0.001
T category				
T2–3	1.000		1.000	
T4	5.475 (2.886–14.137)	0.014	6.889 (2.135–19.559)	0.008
N category				
N0	1.000		1.000	
N1	9.963 (4.472–18.336)	< 0.001	12.559 (6.838–16.945)	< 0.001
M category				
M0	1.000		1.000	
M1-lym^b^	21.958 (11.028–67.559)	< 0.001	16.996 (7.459–70.137)	< 0.001
CRT response				
CR	1.000		1.000	
Not CR	18.965 (13.447–78.775)	0.007	22.598 (9.589–56.779	< 0.001
NS1-BP expression				
High	1.000		1.000	
Low	16.795 (9.984–52.694)	0.002	21.821 (14.470–68.632)	< 0.001

*ESCC* esophageal squamous cell carcinoma, *T* tumor, *N* node, *M* metastases, *CR* complete response

*χ^2^ test

^a^Mean age

^b^Distant lymph node metastases

**Table 3:** Multivariate Cox regression analysis of disease-specific survival

Variables	Learning cohort			Validation cohort		
	HR	95% CI	*P*	HR	95% CI	*P*
NS1-BP expression	2.229	0.929–3.996	0.019	2.518	1.085–5.226	0.010
CRT response	3.271	1.485–7.207	0.003	3.958	1.386–7.667	0.008
N category	1.233	0.586–2.595	0.581	1.663	0.562–3.893	0.329
M category	1.595	0.946–2.691	0.080	1.331	0.667–2.433	0.039

*ESCC* esophageal squamous cell carcinoma, *DSS* disease-specific survival, *HR* hazard ratio, *CI* confidence interval

### NS1-BP suppresses growth and promotes apoptosis of ESCC cells

**Fig. 2:**
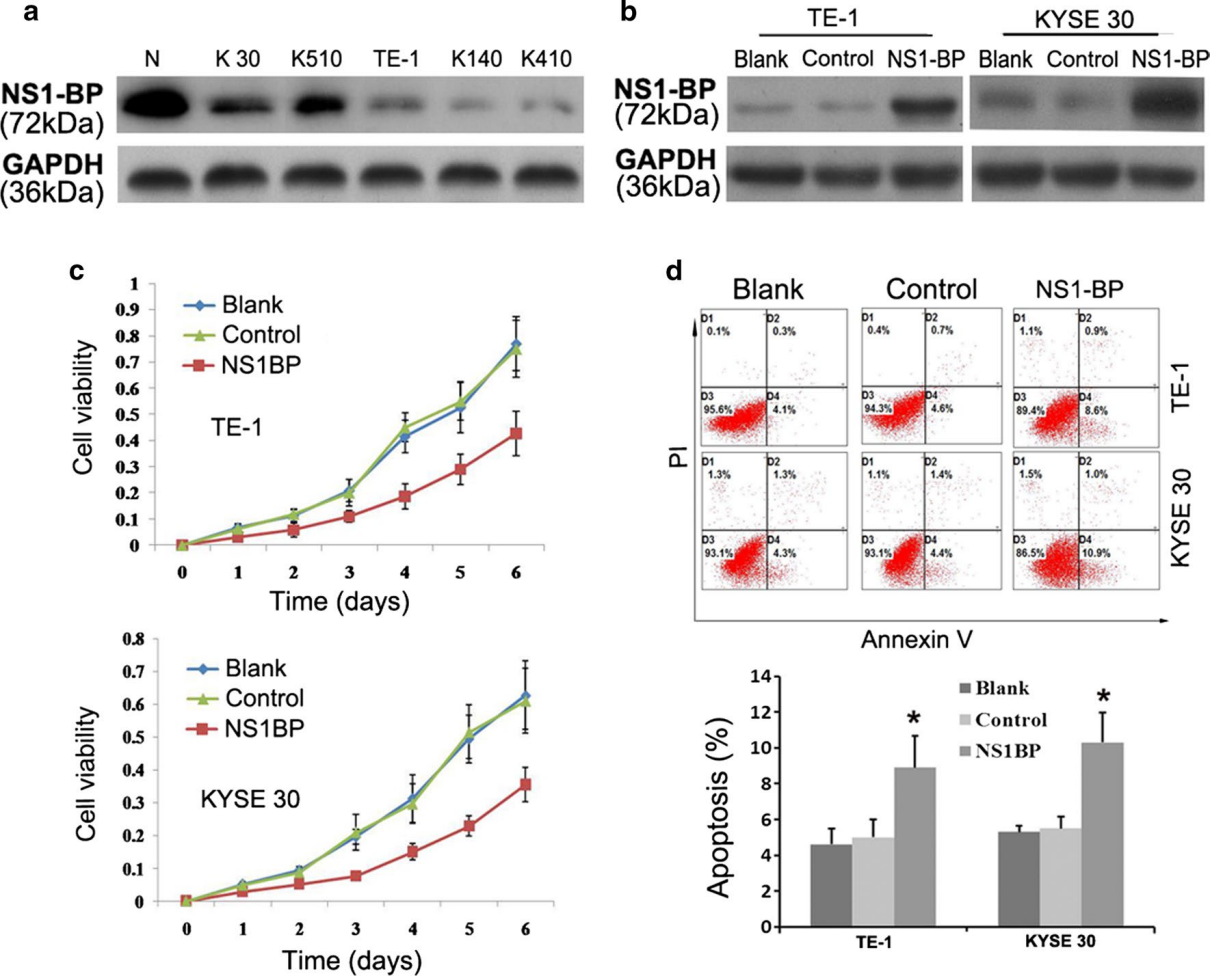
Effect of NS1-BP overexpression on ESCC cell proliferation and apoptosis. **a** Western blot showing that the levels of NS1-BP in five ESCC cell lines (KYSE-30, KYSE-510, TE-1, KYSE-140, and KYSE-410) were lower than in control normal esophageal cells (N). **b** Western blot showing the expression of NS1-BP in stable transfected TE-1 and KYSE-30 cells (TE-1-NS1-BP; KYSE-30-NS1-BP) relative to empty vector control cells (TE-1-Vector; KYSE-30-Vector) and blank control cells (TE-1-Blank; KYSE-30-Blank). Expression was normalized against endogenous GAPDH. **c** Cell growth rate was decreased by ectopic overexpression of NS-BP in TE-1 and KYSE-30 cells, as detected by the 3-(4,5-dimethylthiazol-2-yl)-2,5-diphenyl tetrazolium bromide assay. Results are expressed as mean ± standard deviation (SD) of three independent experiments. **d** NS1-BP promoted tumor cell apoptosis in TE-1 and KYSE-30 cells compared with vector-transfected control cells and blank control cells under normal conditions. Cell apoptotic death events were monitored by annexin V/propidium iodide (PI) staining and flow cytometry assays. The percentage of apoptotic cells is shown as the mean ± SD of three independent experiments. Data represent mean values and SD (*P < 0.05, Student’s *t* test)

All five ESCC cell lines expressed lower NS1-BP levels than the control cells (Fig. 2a). TE-1 and KYSE-30 cells with stable NS1-BP transfection had moderate NS1-BP expression levels (Fig. 2b). The MTT assays showed that ectopic overexpression of NS1-BP inhibited growth of both TE-1 and KYSE-30 cells compared with empty vector control (Fig. 2c). Flow cytometry indicated that NS1-BP significantly promoted apoptosis of TE-1 and KYSE-30 cells (*P *< 0.05, Fig. 2d).

### NS1-BP modulates ESCC radiosensitivity in vitro

**Fig. 3:**
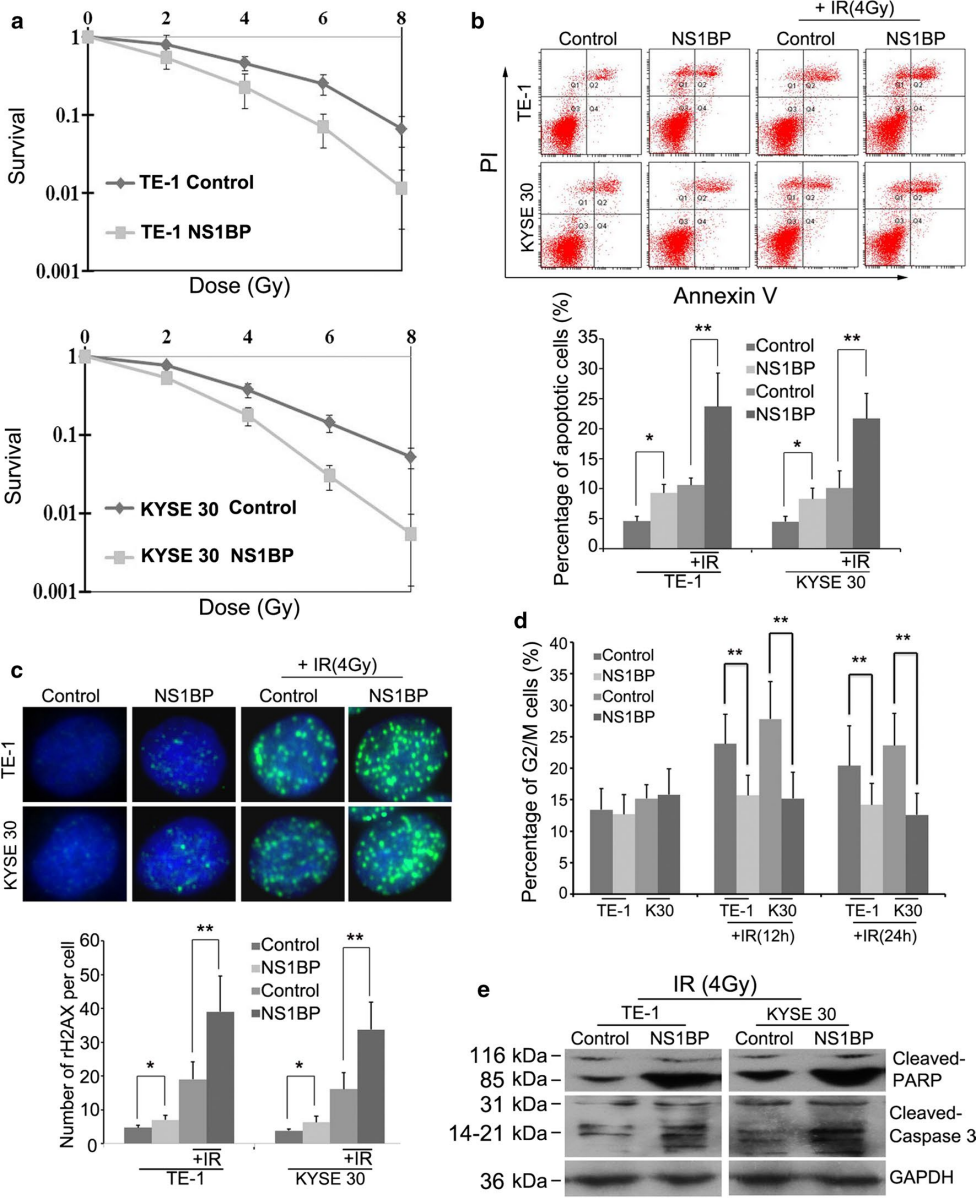
NS1-BP radiosensitizes ESCC cells in vitro. **a** Colony formation assay showing that the survival capacity of ESCC cells ectopically overexpressing NS1-BP was lower than that of control cells after IR (**P < 0.01). **b** Enhanced expression of NS1-BP promoted IR-induced apoptosis in both KYSE-30 and TE-1 cells. Annexin V and propidium iodide staining was used to determine the percentage of apoptotic cells. Data represent mean values and SD (*P < 0.05; **P < 0.01, Student’s *t* test). **c** Ectopic overexpression of NS1-BP the amount of increased unrepaired DNA damages-DNA double strand breaks (DSBs) induced by IR. Cells were subjected to 4 Gy IR and fixed for immunofluorescence 24 h later. Staining for antibodies against γ-H2AX (green) as a measure of DSBs is shown. Quantification of the average number of IR-induced γH2AX foci per cell is also shown (lower panel). Data represent mean values and SD **d** Ectopic overexpression of NS1-BP significantly decreased IR-induced G_2_/M cell cycle arrest after 4 Gy X-ray irradiation in both ESCC cell lines. Panel shows percentage of cells in G_2_/M. **e** Western blot showing that NS1-BP overexpression of increased cleaved caspase 3 and PARP in both TE-1 and KYSE-30 cells exposed to 4-Gy irradiation (*P < 0.05; **P < 0.01, according to Student’s *t* test)

Clonogenic assays showed that NS1-BP-overexpressing ESCC cells had lower clonogenicity than the control cells after IR (Fig. 3a). Compared with the negative controls, over-expression of NS1-BP significantly increased the apoptotic rate of cells upon treatment with IR (Fig. 3b).

**Fig. 4:**
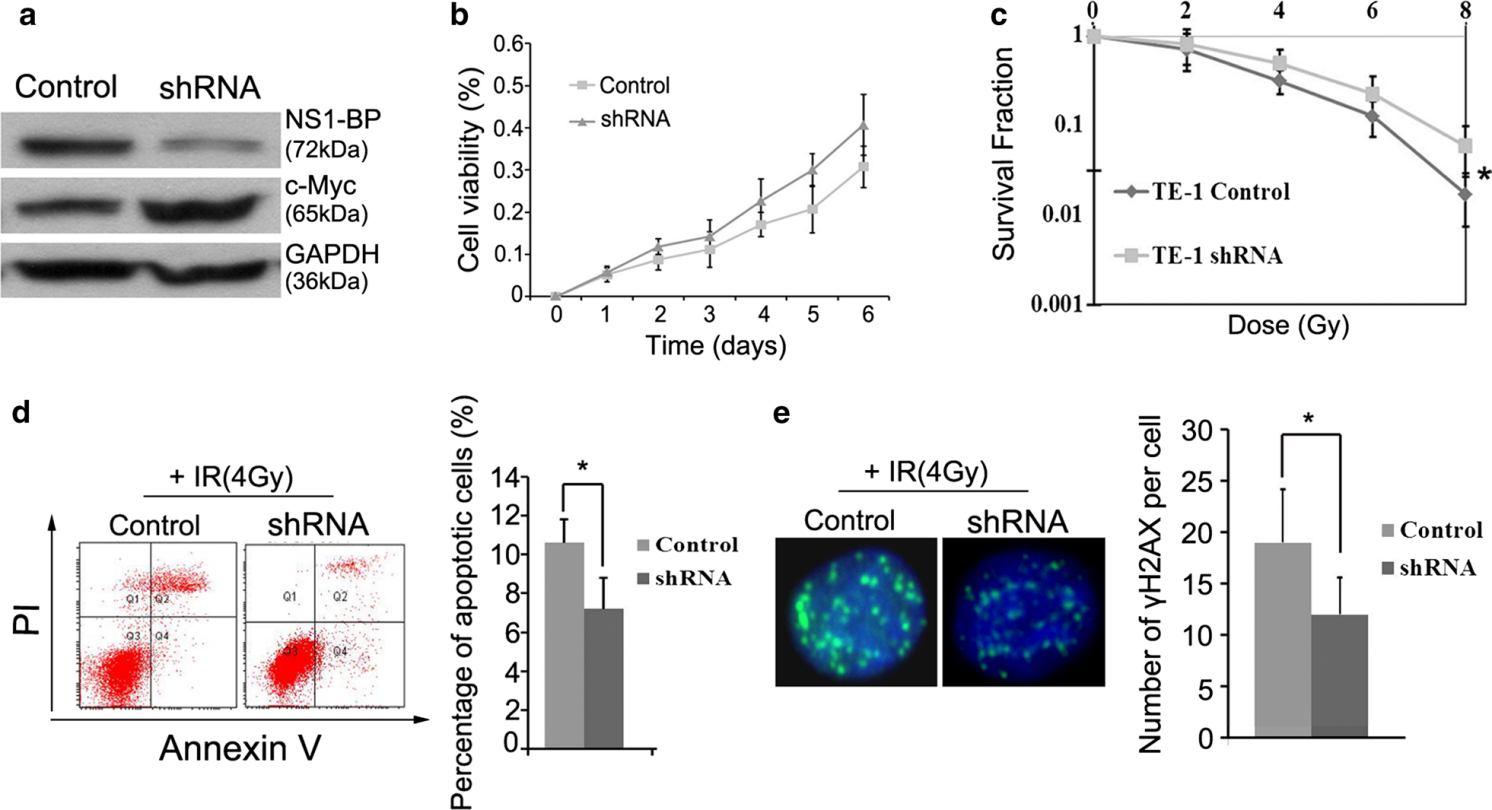
Silencing NS1-BP induces radioresistance of ESCC cells in vitro. **a**, **b** Depletion of NS1-BP decreased c-Myc expression and promoted proliferation of TE-1 cells. **c**–**e** Loss-of-function studies revealed that NS1-BP depletion induced cellular radioresistance (**c**), and attenuated apoptosis (**d**) and unrepaired DNA damage (**e**) induced by irradiation (*P < 0.05, according to student’s t test)

The number of γ-H2AX foci, a hallmark of DNA damages, increased in NS1-BP-overexpressing TE-1 and KYSE-30 cells compared with the corresponding controls 12 h after IR (Fig. 3c). In contrast, depletion of NS1-BP enhanced cellular radioresistance, and decreased the number of apoptotic cells and the amount of unrepaired DNA damages induced by IR (Fig. 4a–e). Over-expression of NS1-BP significantly decreased IR-induced G_2_/M cell cycle arrest after IR (4 Gy) in both ESCC cell lines (Fig. 3d). Western blot analysis showed dramatic increases in cleaved caspase-3 and PARP in NS1-BP-overexpressing ESCC cells compared with the control (Fig. 3d).

### NS1-BP suppresses c-Myc at the transcriptional level

**Fig. 5:**
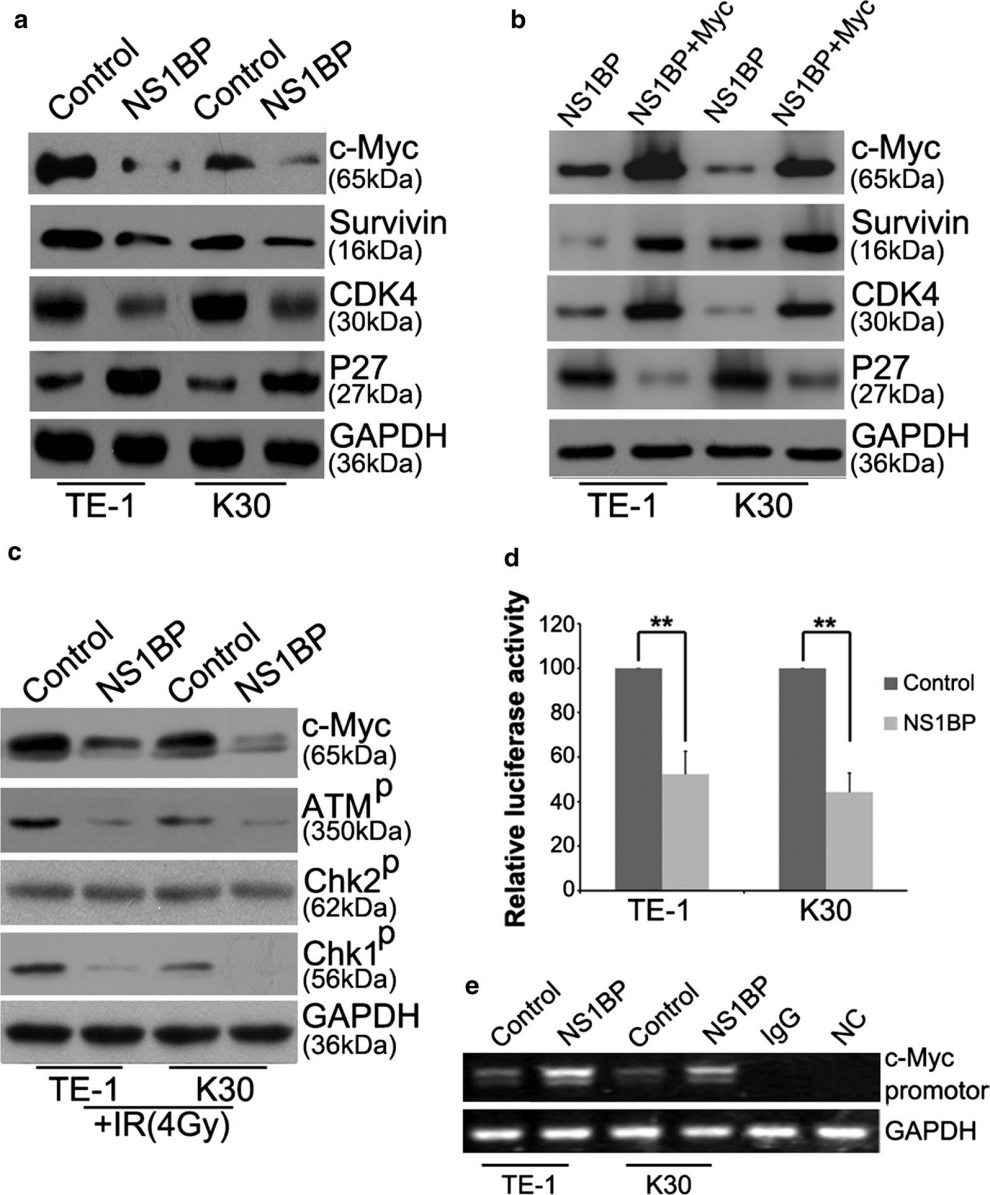
Enhanced expression of NS1-BP suppresses MYC signaling by transcriptionally inhibiting c-Myc. **a** Western blot showing the expression of key downstream factors of the MYC signaling pathway. All data were derived from three independent experiments. **b** c-Myc phenotype reversal in NS1-BP-overexpressing cells restored the expression of survivin, CDK4, and p27. **c** Western blot showing that ectopic NS1-BP expression attenuated irradiation-induced activation of the ATM/Chk1 pathway (*ATM*^*p*^ phosphorylated ATM, *Chk1*^*p*^ phosphorylated Chk1, *Chk2*^*p*^ phosphorylated Chk2). **d** Analysis of luciferase activity. Fragment containing c-Myc promoter region sequence was cloned downstream of the luciferase reporter gene. Plasmids were transfected into empty vectors or NS1-BP stably expressing cells. Renilla luciferase plasmids were co-transfected for normalization. Data are the mean ± SD of three independent experiments (**P < 0.01). **e** ChIP assay on ESCC cellular extracts using NS1-BP antibodies followed by RT-PCR to analyze the associated c-Myc promoter region. (*NC* non-related c-Myc promoter negative control)

Overexpression of NS1-BP inhibited c-Myc expression in both TE-1 and KYSE-30 cells (Fig. 5a). Western blot analysis confirmed that the key proteins required for cell cycle checkpoint control also showed corresponding changes (Fig. 5a). In particular, survivin, a regulator of the mitotic spindle checkpoint, and CDK4 levels were decreased, whereas the inhibitory protein of cell cycle progression p27 increased in NS1-BP-overexpressing ESCC cells (Fig. 5a). The infection of NS1-BP-overexpressing ESCC cells with c-Myc lentivirus to achieve ectopic overexpression of c-Myc restored the levels of survivin, CDK4, and p27 (Fig. 5b).

Western blot analysis showed that IR activated both ATM/Chk1 and ATM/Chk2 signalling (Fig. 5c). Overexpression of NS1-BP significantly inhibited IR-induced ATM/Chk1 phosphorylation, but did not affect Chk2 phosphorylation (Fig. 5c). To further evaluate the mechanism whereby NS1-BP suppresses c-Myc expression, we tested the effect of NS1-BP overexpression using a reporter plasmid containing the *c*-*Myc* promoter upstream of the firefly luciferase gene. Compared with the control cells, luciferase activity was reduced by over 50% in NS1-BP-overexpressing cells (Fig. 5d). ChIP assays indicated that NS1-BP binds to the promoter regions of *c*-*Myc* in both NS1-BP-overexpressing cells and the control cells (Fig. 5e).

### NS1-BP radiosensitizes ESCC cells in vivo

**Fig. 6:**
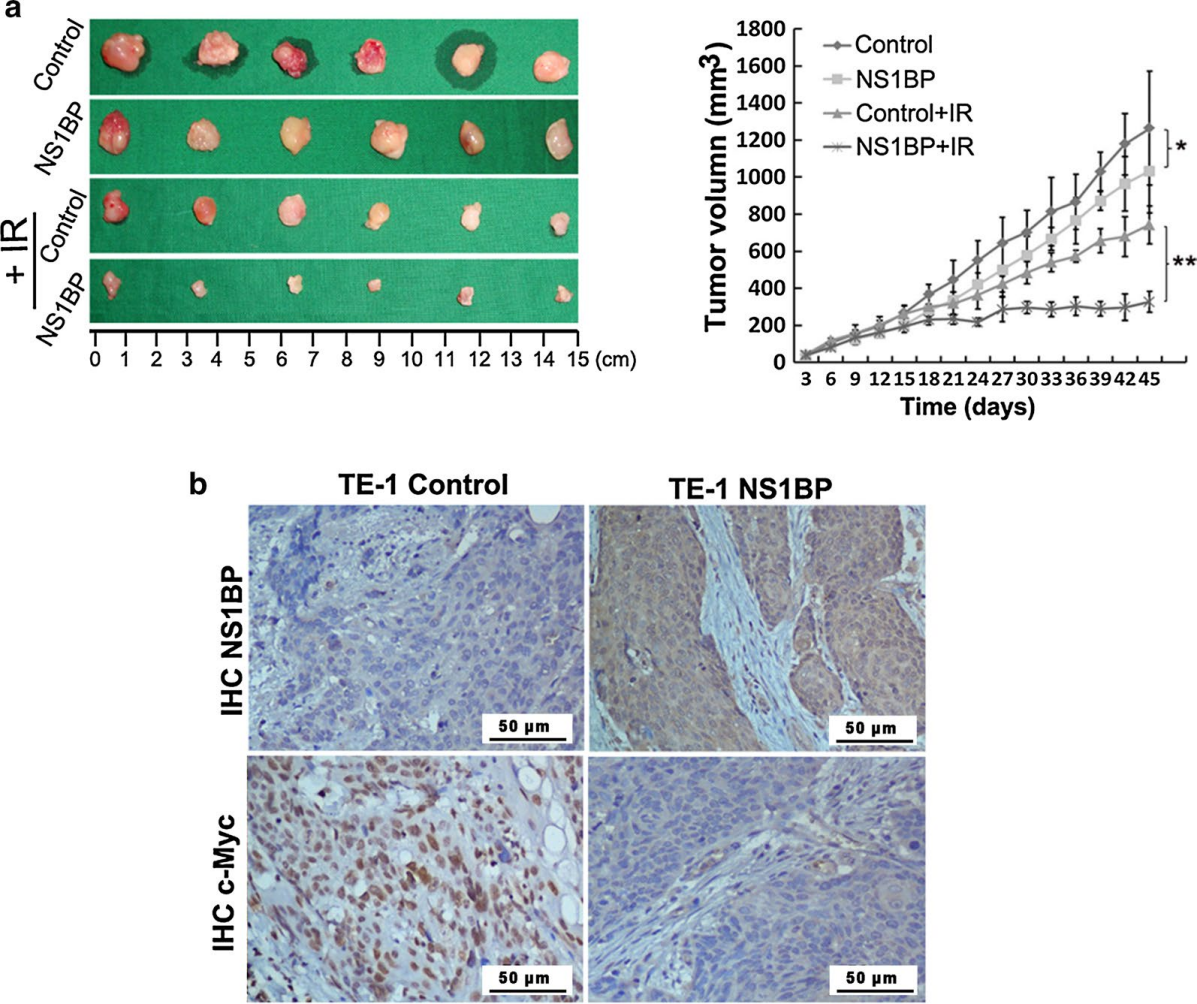
NS1-BP potentiates the therapeutic effect of irradiation on ESCC xenografts. **a** Volumes of tumor xenografts were measured every 3 days with calipers for 36–45 days. NS1-BP inhibited the growth of tumors in the non-treatment groups (control and NS1-BP). The mean tumor volume in the control and NS1-BP groups was 1264.6 ± 240 and 1033 ± 113 mm^3^, respectively (n = 6, *P *= 0.036, Student’s *t* test). After 6-Gy irradiation (control + IR and NS1-BP + IR groups), the mean tumor volume in the NS1-BP group was significantly smaller (326.6 ± 66 mm^3^ versus 742.3 ± 136 mm^3^ in the control; n = 6, *P* = 0.007, Student’s *t* test). Values represent mean tumor volume ± SD (*P < 0.05; **P < 0.01, student’s t test). **b** Representative images show tumor xenografts in null mice with TE-1-Control and TE-1-NS1-BP cells. Immunohistochemical staining of NS1-BP and c-Myc of tumor sections excised from mice. Lower c-Myc expression was seen in NS1-BP-overexpressing cells than in TE-1 vector control cells

We next investigated whether NS1-BP could affect ESCC cellular response to IR in vivo. NS1-BP-overexpressing TE-1 cells and the control cells were inoculated into female athymic nude mice. When tumors reached a size of at least 180 mm^3^, the xenografts were irradiated at a dose of 6 Gy (2 Gy × 3 fraction). Consistent with the in vitro results, NS1-BP inhibited growth of TE-1 xenografts (Fig. 6a). NS1-BP also significantly delayed tumor xenograft growth treated with IR (Fig. 6a). Immunohistochemistry showed lower c-Myc expression in NS1-BP-overexpressing cells than the TE-1 vector control cells (Fig. 6b).

### c-Myc levels negatively correlate with NS1-BP expression

**Fig. 7:**
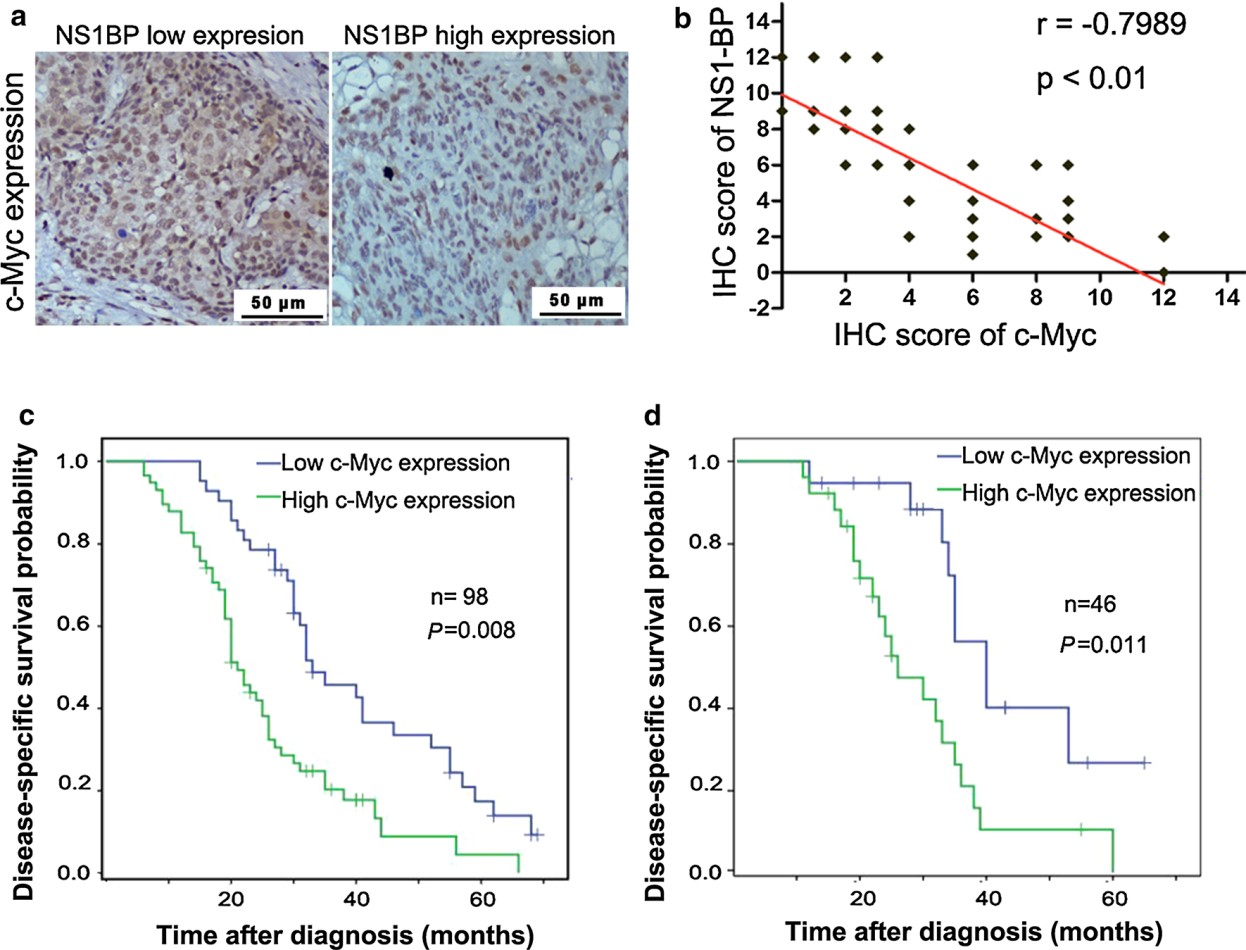
c-Myc negatively correlates with NS1-BP expression in ESCC. **a** Immunohistochemical staining of c-Myc in ESCC samples. Representative images showing higher expression of c-Myc in samples with low NS1-BP levels, and lower expression of c-Myc in samples with high NS1-BP levels. **b** c-Myc expression was inversely correlated with NS1-BP levels in 144 ESCC samples. The correlation was determined with linear regression lines and Pearson’s correlation significance (Pearson R = − 0.7989, **P < 0.01). **c**, **d** Upregulation of c-Myc was significantly associated with poor prognosis of ESCC patients. Kaplan–Meier plots show disease-specific survival in 98 ESCC patients in the training cohort (**c**) and 46 ESCC patients in the validation cohort (**d**) according to c-Myc expression levels in the primary tumor (*P* = 0.008 and 0.011, log-rank Table 1 Association between clinicopathologic factors and NS1-BP expression in esophageal squamous cell carcinoma (ESCC) patients

NS1-BP expression was inversely correlated with c-Myc levels (Pearson *r* = − 0.7989, *P *< 0.01, Fig. 7a, b). Higher c-Myc levels were also associated with a shorter DSS (Fig. 7c, d).

## Discussion

NS1-BP reportedly inhibits pre-mRNA splicing, and regulates influenza A viral gene expression and replication [13]. However, little is known about its expression and importance in human cancers. In the present study, we demonstrated that NS1-BP downregulation is positively correlated with chemoradiotherapeutic resistance and a shorter DSS. Furthermore, our in vitro and in vivo experiments revealed that ectopic restoration of NS1-BP enhanced ESCC radiosensitivity by suppressing c-Myc signalling at the transcriptional level. Taken together, our findings highlight the fundamental roles of NS1-BP in ESCC, and suggest its potential application as a prognostic predictor of ESCC.

Genetic and epigenetic comparisons of normal tissue and tumor tissues are arguably the most effective way to identify tumor-related genes [24, 25]. In the present study, we first demonstrated that NS1-BP expression was lower in ESCC tissues than paired adjacent normal esophageal mucosal tissues. In both cohorts of ESCC patients, NS1-BP downregulation was positively correlated with chemoradiotherapeutic resistance and a shorter DSS. Thus, NS1-BP levels could predict the outcome of ESCC patients treated with definitive chemoradiotherapy, so examination of NS1-BP expression by immunohistochemistry may be used to identify ESCC patients who are resistant to chemoradiotherapy.

The clinical value of a molecular marker for cancer is related to its biological function [26–28]. Cancer cells display a variety of deregulate homeostatic signals [29]. Moreover, undue proliferation of tumor cells and blocking of the apoptotic pathway contribute greatly to tumorigenesis [29–31]. We showed that ectopic restoration of NS1-BP expression suppressed cell proliferation and tumorigenicity, and promoted apoptosis of TE-1 and KYSE-30 cells. These findings suggest that NS1-BP downregulation participate in ESCC development and progression.

Radiotherapy is a critical part of the management of locally advanced ESCCs. Resistance to IR is a major cause of cancer treatment failure [32, 33]. On this basis, we further investigated whether NS1-BP influenced radiosensitivity in ESCC cells. Our results clearly showed that overexpressing NS1-BP could substantially increase cytotoxic response to IR both in vitro and in vivo. Ectopic overexpression of NS1-BP significantly enhanced IR-induced apoptosis, and remarkably increased the amount of unrepaired DNA damages. NS1-BP also enhanced the sensitivity of ESCC cells to cisplatin (data not shown). Moreover, NS1-BP effectively abrogated IR-induced G_2_/M cell-cycle arrest.

Apoptosis and unrepaired DNA damages are considered major cell death mechanisms after IR in solid tumors [34, 35]. IR-induced cell-cycle arrest mainly contributes positively to cell survival in response to radiation [36]. Thus, our results strongly suggest that NS1-BP downregulation in ESCC confers radioresistance by preventing cells from undergoing apoptosis, and maintaining IR-induced G_2_/M cell-cycle arrest. Perconti and colleagues [37] reported that NS1-BP could inhibit the proliferation of HeLa cells and suppress *c*-*Myc* at the transcriptional level. Consistently, we found that ectopic overexpression of NS1-BP significantly decreased c-Myc protein levels, likely via direct interaction between NS1-BP and *c*-*Myc* promoter sequence. In our clinical ESCC tissues, we also found that c-Myc expression was inversely correlated with NS1-BP levels, and that high c-Myc expression predicted a poor DSS for ESCC patients. These results suggest that c-Myc is a downstream signalling molecule of NS1-BP.

c-Myc regulates 15% of all human genes, and plays an important role in many biological processes that are associated with both apoptosis induction and cell-cycle arrest [38, 39]. As expected, we detected decreased levels of CDK4 and survivin, which are downstream factors of c-Myc that regulate cell cycle and cellular proliferation, following NS1-BP overexpression. Similarly, a major cell cycle inhibitory protein, p27, was increased following c-Myc depletion. In response to IR-induced DNA damage, ATM is rapidly activated by c-Myc and other factors, which, in turn, activates Chk1 and Chk2 [36]. In accordance with previous studies, we found that IR activated both the ATM/Chk1 and ATM/Chk2 signalling pathways in ESCC cells. Interestingly, enhanced expression of NS1-BP significantly inhibited IR-induced ATM/Chk1 phosphorylation, but had no impact on Chk2 phosphorylation. ATM/Chk1 activation induces both G_1_ arrest and G_2_/M arrest, whereas ATM/Chk2 activation mainly induces G_1_ arrest. The G_1_ checkpoint is defective in most cancer cells because of mutations/alterations of its key regulators, whereas activation of the G_2_ checkpoint is rarely impaired in cancer cells. Therefore, abrogation of the G_2_ checkpoint often sensitizes cancer cells to IR [36, 40]. Our findings showed that NS1-BP could decrease IR-induced G_2_/M cell-cycle arrest by suppressing the c-Myc/ATM-/Chk1 pathway and sensitizing ESCC cells to IR. Many other downstream targets of c-Myc, and indeed, molecules other than c-Myc, are implicated in the sensitivity of cancer cells to IR. Additionally, the interactions between NS1-BP and the *c*-*Myc* promoter have not been delineated in detail. Further studies are required to understand the mechanism of NS1-BP regulation of c-Myc.

## Conclusions

In summary, decreased NS1-BP expression predicts poor treatment responses to radiotherapy in patients with advanced ESCC, possibly by suppressing the c-Myc signalling pathway at a transcriptional level.

## Abbreviations

ESCC: esophageal squamous cell carcinoma
DSS: disease-specific survival
IR: ionization radiation
CR: complete response
PR: partial response
TNM: tumor node metastases
ChIP: chromatin immunoprecipitation
